# Azathioprine Induced Pancytopenia in a Patient with Vogt-Koyanagi-Harada Disease: A Case Report

**DOI:** 10.31729/jnma.7867

**Published:** 2022-10-31

**Authors:** Sagun Khatri, Saugat Khatri, Sachit Regmi, Sulav Pyakurel, Sanjeet Bhattarai, Pankaj Barman, Dilasha Manandhar, Abhi Kumar Singh

**Affiliations:** 1 B.P. Koirala Institute of Health Sciences, Dharan, Sunsari, Nepal; 2 Nepal Mediciti, Nakhkhu Patan, Lalitpur, Nepal; 3 Nepal Medical College and Teaching Hospital, Jorpati, Kathmandu, Nepal; 4 Postgraduate Institute of Medical Education and Research, Madhya Marg, Chandigarh, India

**Keywords:** *case reports*, *pancytopenia*, *steroid*

## Abstract

Vogt-Koyanagi-Harada disease is a multisystem autoimmune inflammatory disorder that affects the eyes, ears, skin, and the nervous system. It is a rare disease that mainly affects Asian, Hispanic, and Middle Eastern populations. Systemic steroids and immunosuppressants are frequently used to treat autoimmune diseases like this. Despite the fact that they reduce morbidity, immunosuppressants can have a number of side effects and are difficult to use, particularly when treating uncommon autoimmune illnesses. We present a case of a 56-year-old man who visited our health facility complaining of increased tears in both eyes along with bilateral blurring of vision. He was subsequently identified as having Vogt-Koyanagi-Harada disease. After prednisolone and methotrexate failed to have the desired effect, he was treated with azathioprine, which caused pancytopenia, and manifested as fever with positive blood culture for a coagulase-negative staphylococcus infection.

## INTRODUCTION

Acute eye symptoms without any preceding trauma or events have many differential diagnoses. One of them is Vogt-Koyanagi-Harada (VKH) disease accounts for 7-8% of all patients with uveitis in Japan.^1^ VKH is a multisystem autoimmune disorder in which the majority of individuals experience bilateral ocular inflammation along with neurologic, auditory, and/or integumentary symptoms and signs. Given the lack of confirmatory tests and the fact that the condition is only identified based on a constellation of symptoms, the disease can be challenging for doctors and even more so for the patients. Additionally, it becomes more difficult when immunosuppressive medicine is ineffective or when a patient encounters potentially fatal side effects from their treatment.

## CASE REPORT

A 56-year-old male presented with a history of bilateral blurring of vision which was acute in onset, gradually progressive, and was not accompanied by any pain. However, it was associated with occasional mild tearing of bilateral eyes. Additionally, he also mentioned the presence of floaters and flashes over bilateral eyes time and again. Although he did not have any headaches, he did complain of occasional minor photophobia. On examination, there was no redness of the eyes or diplopia. Eyelid enlargement, drooping, or foreign body sensation in either eye was absent. There was no previous history of penetrating ocular trauma, surgery, or any other eye diseases.

**Figure 1:**
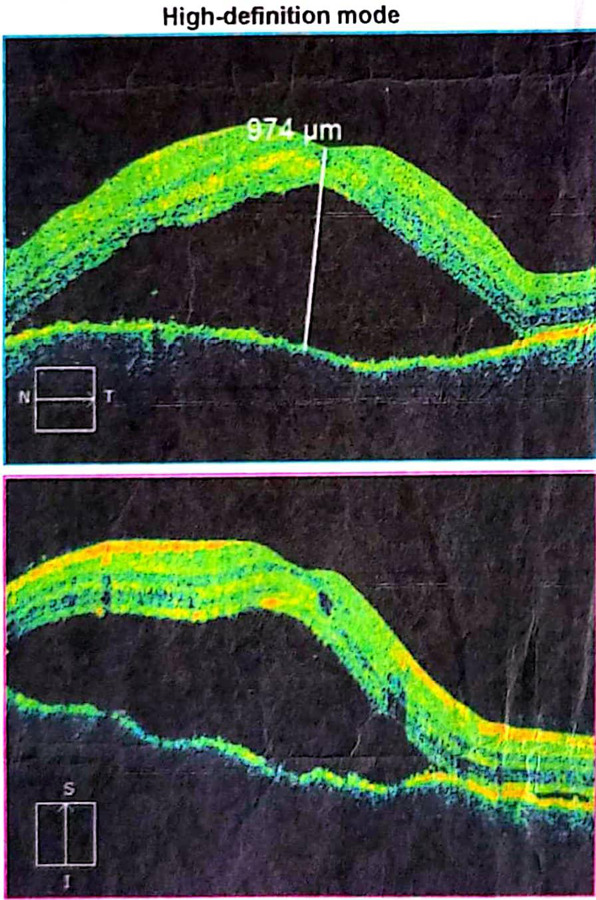
An OCT showing the macular fluid collection.

During this period, he also detected growing symmetrical patchy macular depigmentation over the temporal region, dorsum of his back, shoulders, and both pairs of his feet. On examination, he had a vision of 6/36 in his right eye and 6/60 in his left eye. His intraocular pressures (IOP) were normal with 12 mm Hg in his right eye and 15 mm Hg in his left eye. Also, his Optical Coherence Tomography (OCT) showed subretinal fluid and features suggestive of central serous chorioretinopathy (Figure 1).

**Figure 2:**
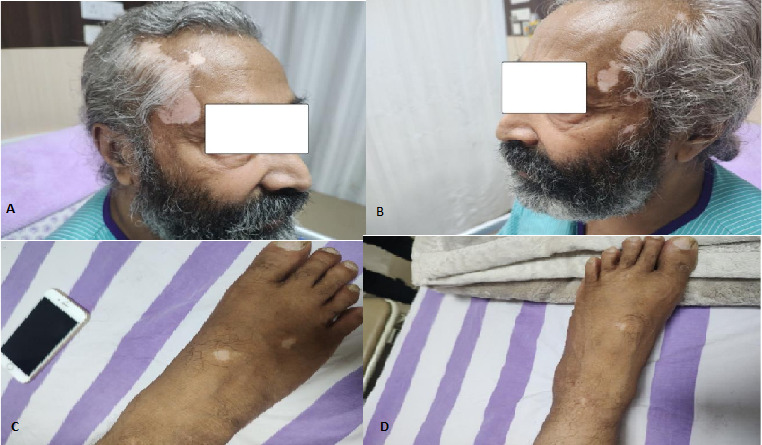
Vitiligo on A) Right temporal-parietal region, B) Left temporal-parietal region, C) Dorsum of right foot, D) Dorsum of the left foot.

**Figure 3:**
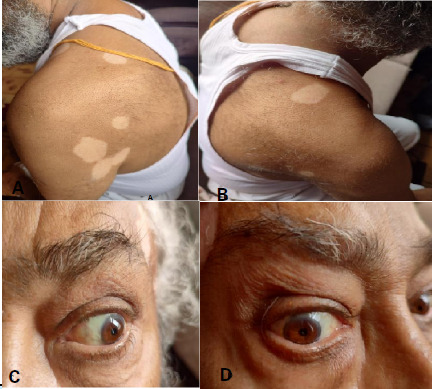
Vitiligo on A) Left shoulder and posterior arm, B) Right shoulder and posterior arm, C) Left perilimbal vitiligo, D) Right perilimbal vitiligo.

After dermatological consultation, he was diagnosed with vitiligo (Figure 2). On further examination, he had bilateral perilimbal vitiligo (Figure 3). The lesions were not itchy or uncomfortable. He had no history suggestive of diabetes mellitus, hypertension, tuberculosis or any malignancy. No previous history of neurological condition existed. However, he had received treatment for psoriasis in an institute 21 years ago.

He smoked 5-10 cigarettes per day for around 25 years which accounts for approximately 10 pack-years but had left for 5 months. He used to take marijuana on a regular basis in the past and consumed alcohol occasionally. He was using spectacles as an eye aid with the power of +1.5D.

After clinically ruling out other eye diseases and given the nature of his symmetric skin and ocular lesions, he was eventually diagnosed with VKH. He was started on oral steroid therapy, prednisolone 75 mg with a plan to continue the same dose for 6 months. However, despite the use of steroids for 6 weeks, blurring of vision persisted and he had progression of vision loss with his right eye vision being 6/12 on follow-up, necessitating the use of an oral immunosuppressive drug (methotrexate) once a week for 13 weeks. However, it affected his liver function test. Additionally, there was no discernible clinical improvement, so he was recommended to cease methotrexate therapy and instead begin 50 mg of azathioprine (AZA) every day. He was advised to follow up on a weekly basis with complete blood count reports. This helped with the clinical improvement of his blurring of vision.

Despite his advice for weekly visits to monitor the side effects of AZA, he presented after 2 months with a history of high-grade fever, with a maximum temperature of 106°F, along with chills and rigors. Additionally, he reported experiencing two episodes of vomiting that were non-bilious and bloodless and contained food particles. No history of shortness of breath, jaundice, or stomach or chest discomfort was present. He had normal bladder and bowel movements. There was no history suggestive of unexpected weight loss, abnormal body movements or loss of consciousness.

He was admitted to our health facility and on further workup, his total leukocyte count (TLC) was 1930 cells/mm^3^, red blood count (RBC) was 2.5 million/mm^3^, packed cell volume (PCV) was 24.6%, haemoglobin was 7.9 gm% and platelet count was 95000 cells/mm^3^. He also had a mild derangement of his liver function test (LFT) and renal function test (RFT). A coagulase-negative staphylococci (CONS) infection was detected on his blood culture. He had three pints of Packed RBC transfusions done throughout his hospital stay in addition to intravenous (IV) antibiotics and other supportive medications. Transfusion periods were uneventful. His TLC grew to 3530 cells/mm^3^, RBC to 2 million/mm^3^, PCV to 28.9%, hemoglobin to 9.2 gm%, and platelet count to 120,000 cells/mm^3^ at the time of discharge. Bone marrow aspiration was not done for pancytopenia due to the obvious aetiology being the use of AZA. He was discharged on oral cefixime, methylcobalamin, and topical steroids. He was also advised for a regular follow-up with CBC, and LFT, and if favourable, was planned for initiating a different immunosuppressant in the next visit.

## DISCUSSION

A genetically vulnerable person may develop VKH, which primarily attacks the melanin-containing cells found in the skin, meninges, ears and eyes. Though it is considered rare in the Nepalese population, there have been several cases of VKH reported. Being a multi-systemic disease, it can have significant morbidity in a person's life including significant visual loss, but timely identification and treatment can minimise ocular morbidity. Due to the rarity of VKH, no definitive confirmatory test has been developed, and the diagnosis is made based on a combination of signs and symptoms.^2^ Since our patient did not have any neurological symptoms, he was diagnosed with incomplete Vogt-Koyanagi-Harada disease based on the diagnostic criteria for VKH.^3^

Immunosuppressive drugs is the mainstay of treatment for VKH, as they are for the majority of other autoimmune illnesses. However, adverse effects from these medications are not uncommon. While preventing the progression of vision loss, immunosuppressive drugs may cause nephrotoxicity, hepatotoxicity, adrenal suppression, systemic infection, hypertension and bone marrow suppression.^4^ Our patient was initially treated with prednisolone 75 mg for 6 weeks which didn't show any clinical improvement after which he was put on methotrexate for 13 weeks which resulted in hepatotoxicity and had to be stopped. Azathioprine was started but it eventually led to a life-threatening infection of CONS secondary to pancytopenia.

AZA is a purine analogue that, through the actions of the enzymes hypoxanthine-guanine phosphoribosyltransferase (HPRT) and thiopurine methyltransferase (TPMT), transforms into its active metabolites, 6-mercaptopurine (6-MP) and 6-thioguanine (6-TGN). It then prevents the production of purines. Its metabolites stop division by integrating with the deoxyribonucleic acid (DNA) that is duplicating. The majority of AZA's toxic and immunosuppressive effects may potentially be mediated by its metabolites. It is metabolized in the liver and eliminated through the kidneys, which makes it more hazardous in cases of renal failure.^5^

Bone marrow suppression frequently accompanies AZA/6-MP use. Around 5-10% of individuals have myelotoxicity after receiving AZA/6-MP, depending on the dosage. The likelihood of bacterial infections rises with bone marrow depression.^6^ Other side effects include anorexia, nausea, vomiting, and diarrhoea. Most frequently seen in 5-15% of treated people are brief, mostly asymptomatic increases in blood enzymes, primarily ALT and AST.^7^ Our patient was not under any xanthine oxidase inhibitors which are known to increase the toxicity of AZA.^8^ However, he had a systemic infection with CONS and was successfully treated with IV antibiotics.

Immunosuppressants are still crucial for treating and halting the course of autoimmune disease despite their side effects and long-term consequences. A proper follow-up with blood work and LFT is essential. However, patients are frequently non-adherent to follow-up. This is a big challenge for us as medical professionals, particularly when treating uncommon diseases.

The main takeaway from our case report is the prevalence of uncommon diseases in a country like Nepal where the majority of healthcare issues involve infectious diseases and rare diseases are less frequently diagnosed. Since diseases have overlapping features, we generally but not always, lean towards a simple diagnosis. We firmly believe that science is a young subject, it is growing every day and we must grow with it. The morbidity of rare diseases should not be disregarded. The problems of treating such uncommon diseases exist, but they may be solved with a systematic approach.
